# News and Coming Events

**Published:** 1907-11-02

**Authors:** 


### NEWS AND COMING EVENTS.

It has been decided to increase the accommodation of the Essex and Colchester Hospital by the addition of a children's ward at an estimated cost of £2,500.

Mr. Archibald Ramsden, head of the well-known firm of Archibald Ramsden, Ltd., piano dealers, Park Row, Leeds, has given a donation of £1,000 to the funds of the Leeds Workpeople's Hospital Fund.

At the forthcoming Franco-British Exhibition to be held in London early next year, the Committee of Class 16 (medicine and surgery) will exhibit a complete set of medical and surgical books and reviews which have been published in France during the last five years.

Dr. Alexander Macphail, Professor of Anatomy at St. Mungo's College, Glasgow, was given a complimentary dinner and a handsome presentation by a hundred medical men of that town on the occasion of his relinquishing his appointment for that of Lecturer in Anatomy at Charing Cross Hospital, London.

A brass tablet has just been unveiled at the North Infirmary, Cork, to commemorate the services of one of the most generous supporters of the institution. It bears the following inscription : "This tablet has been erected by the Trustees of the North Charitable Infirmary, Cork, in grateful recognition of the munificent generosity of James O'Malley M'Donnell, M.D.,M.Ch., F.R.C.S., lieutenant-colonel, Bengal Army, who, in pursuance of a laudable desire to provide a refuge in sickness for those who have seen better days, has endowed two beds in this institution at a cost of £2,000.

In Faith and Hope the world will disagree,
But all mankind's concern is charity."

The usual monthly meeting of the Medical Sickness, Annuity, and Life Assurance Society was held at 6 Catherine Street, Strand, London, W.C. The accounts presented showed that during the first eight months of this year the operations of the Society had been greater than in any similar period of its working. Since January 1 last nearly £9,000 has been distributed to the members in the shape of weekly sick pay, and good cash bonuses have been given to those who reached the limiting age of sixty-five; yet the funds, which now amount to over £200,000, have received a substantial increase, as the annual income of the Society is considerably larger than its outgo. Prospectuses and all particulars may be had on application to Mr. F. Addiscott, Secretary, Medical Sickness and Accident Society, 33 Chancery Lane, London, W.C.

An Exhibition in aid of the funds of the Special Appeal Committee of the Royal Waterloo Hospital will be opened on November 4 by H.R.H. Princess Louise, Duchess of Argyll. An exhibition of works of art (including pictures by old and modern masters) will be held at the Doré Gallery, 35 New Bond Street, from November 4 to 21 inclusive, in aid of the funds of the Special Appeal Committee of the Royal Waterloo Hospital for children and women. The programme is to include daily afternoon concerts, tea, etc. Through the kindness of Lady Evelyn Ewart, the committee have obtained the co-operation of the Ladies' Kennel Association, whose members are working assiduously for this scheme. All information can be obtained from Colonel M. Graham and E. St. C. Bolton, Esq., hon. secretaries, at the Doré Gallery.

In these days of pageants the old-fashioned bazaar has smartened up into something rather more picturesque and less humdrum. At least "Ye Olde Camberwell Fayre," held on October 29, 30, and 31, in aid of the King's College Hospital Removal Fund, transformed the Camberwell Baths into a scene of animation and bustle such as seldom enlivens a bazaar. Under an exceedingly strong organising Committee, representing both sides of the river, a wealth of amateur and professional assistance was obtained, and the vocal performances of the local member of Parliament must also have been a great attraction. The Fayre was opened by T.R.H. the Duke and Duchess of Connaught, whose interest in all charitable enterprises is no new story. The Duke is president of the hospital, and his speech in reply to that of Dr. Macnamara, showed his keen interest in the removal to Camberwell. The stalls of the Fayre represented all sections of local opinion, political and religious, to each of which a distinguishing costume of some period of the seventeenth and eighteenth centuries was assigned. So numerous were the staff of patched and bewigged workers, and so excellent the entertainments in "Richardson's show," that surely never did Camberwell Fair prove such a rollicking success before, or it would never have been abandoned, as it was, after the last show in 1855. There can be little doubt that the Removal Fund will benefit in no niggardly sum from this highly successful Fayre.

Princess Louise has graciously consented to open the wing of the Leicester Infirmary on November 5 next. The building when fitted and furnished will have cost £20,000. Her Royal Highness has also consented to open on the first day a three days' bazaar and sale of work in aid of the funds for the new Nurses' Home to be held in the New Wing at the close of the opening ceremony. On the second and third days the bazaar will be opened by the Countess of Lonsdale and Sir Edward Wood (Mayor of Leicester) respectively. The architect's estimate for building and furnishing the Home, which will contain upwards of one hundred bedrooms, is £20,000.

At the first general meeting of the Therapeutical and Pharmacological Section of the Royal Society of Medicine, held on October 22, in the Apothecaries' Hall, the President of the Section, Dr. Burton Brown, read a short paper containing an account of the Therapeutical Society—its origin, rise, and the work it accomplished, and of the reasons which led to its dissolution and its reconstruction as the Therapeutical and Pharmacological Section of the Royal Society of Medicine. A vote of thanks having been unanimously accorded to Dr. Burton Brown, Dr. Robert Hutchison opened a discussion on "The Scientific Treatment of Dyspepsia." He emphasised in particular the need for more accurate classification of cases of dyspepsia, if progress was to be made. He gave it as his opinion that although diet was of use in the treatment of dyspepsia, the most efficient means was to be found in drugs; of these he recommended especially bismuth and the alkalies. Ferments he seldom prescribed. The most difficult cases to treat were those where there was motor insufficiency; of the benefit of electrical treatment he was still doubtful. In the ensuing discussion Dr. Habershon, Dr. Herschell (who advocated in particular the use of electrical treatment), Dr. Cameron, and Dr. Gray Duncanson took part. A vote of thanks to Dr. Hutchison concluded the meeting.

